# (*in vivo* Gastrocnemius Muscle) Tendon Ratio in Patients with Cerebral Palsy

**DOI:** 10.2174/1874325001711010577

**Published:** 2017-07-28

**Authors:** Muhammad Naghman Choudhry, Haris Naseem, Ihsan Mahmood, Adeel Aqil, Tahir Khan

**Affiliations:** 1 Royal Manchester Children's Hospital, Upper Brook Street, Manchester, M13 9WL, U.K; 2 James Cook University Hospital, Marton Road, Middlesbrough, TS4 3BW, U.K; 3 Pinderfields Hospital, Aberford Road, Wakefield,WF1 4DG, U.K; 4 Royal National Orthopaedic Hospital and Institute of Orthopaedics, University College London, Brockley Hill, Stanmore,HA7 4LP, U.K

**Keywords:** Cerebral Palsy, Gastrocnemius Muscle, Gastrocnemius Lengthening, Muscle-tendon junction, Gastrocnemius, Tendon

## Abstract

**Background:**

The position of the gastrocnemius tendon in relation to the leg length may be different in children with cerebral palsy as compared to normal children. The palpation of muscle bellies or previous experience of the operating surgeon is employed to place the surgical incision for lengthening of the gastrocnemius aponeurosis. Inaccurate localisation may cause incorrect incisions and a risk of iatrogenic damage to the vital structures (i.e. sural nerve).

**Objectives:**

The aim of our study is to compare gastrocnemius length *in-vivo* between paretic and unaffected children and create a formula to localise the muscle–tendon junction accurately.

**Methods:**

10 children with di/hemiplegia (range 2-14y) were recruited. None of them had received any conventional medical treatment. An equal number of age/sex matched, typically developing children (range 4-14y) were recruited. Ultrasound scanning of the gastrocnemius muscle at rest was performed to measure the length of gastrocnemius bellies. We also measured the heights and leg lengths in all the children.

**Results:**

The gastrocnemius medial muscles were shorter in Cerebral Palsy children when compared to similar aged normal children. In cerebral palsy children, the gastrocnemius muscle and leg ratio ranged between 35 to 50% (average ratio of 45%).

**Conclusion:**

Using these figures, we created an average percentage for gastrocnemius muscle length that may be used clinically to identify the tendon for open/endoscopic lengthening and also to make simple and accurate localisation of gastrocnemius muscle-tendon junction for surgical access. This decreases the length of the surgical incision and may reduce the risk of iatrogenic injuries.

## INTRODUCTION

Intra-muscular (myofascial) lengthening or Botulinum injection of the gastrocnemius muscle is a commonly performed procedure to manage the spasticity or shortening of muscle-tendon unit. This usually leads to improved mobility in children with Cerebral Palsy (CP).

Tashjian *et al.* (2003), Pinney *et al.* (2004), and Carl and Barret (2005) have produced clinical guidelines to locate the gastrocnemius tendon based on cadaveric dissections in adults with equinus contracture undergoing Strayer recessions [1-3]. More recently, Elson *et al.* have described palpable bony landmarks to locate gastrocnemius tendon [4]. These studies were performed on normal adults either *in vivo* or on cadavers. It is possible that the position of the gastrocnemius tendon relative to the calcaneus and fibular head distance may be different in children with CP. Kucharski *et al.* have recently reported use of ultrasound scanning of the gastrocnemius at the time of surgery to localise the surgical incision [5].

The muscle fascicle lengths in children with diplegia are shortened as compared to non-paretic legs [6]. The effect of spastic cerebral palsy on the in-vivo gastrocnemius muscle fascicle length is not known. It has been postulated that the medial gastrocnemius is short and small in the paretic limb of children with Spastic Hemiplegia [6].

The present study aims to compare gastrocnemius muscle length in-vivo between paretic children and typically developing children in order to better understand difference in gastrocnemius muscle length between the two groups. The data has been used to suggest a formula for better localisation of gastrocnemius muscle when treating children with CP.

## MATERIALS AND METHODS

### Participants

10 children with di/hemiplegia (seven females and three males; mean age 8y 7mo, range 2-14y) were recruited through the outpatient orthopaedic clinic of the Royal Manchester Children's Hospital in Greater Manchester. None of these children had received any form of conventional medical treatment (such as surgery or botulinum toxin injection) before participation in the study. An equal number of age / sex matched, typically developing children (mean age 9y 1mo, range 4-14y) were recruited from the local schools. Parents of the children provided consent to their participation in the study.

There were 2 children with right hemiplegia and the other 8 suffered from diplegia *i.e.* both legs involved.

### Procedure

Sagittal-plane ultrasound scanning of the gastrocnemius muscle at rest was performed with the use of either of two scanning systems: ALOKA SSD-5000 system, with a 10MHz scanning frequency; ALOKA Co. Ltd, Tokyo, Japan; or MyLab25 system, with a 12MHz scanning frequency; Esaote SpA, Genova, Italy). Participants laid prone on an examination plinth with their feet hanging from its edge during scanning.

The dominant leg of the typically developing children (determined by Coren's footedness inventory) and dominant leg of children with diplegia (that showed a smaller range of motion in the dorsiflexion direction) were scanned. In the two hemiplegic children, the affected right legs were scanned. Several ultrasound scans were obtained from the middle region of the gastrocnemius medialis (GM) and gastrocnemius lateralis (GL) muscles at the proximal, middle, and distal sections of each muscle head. All scans were analyzed in the Image J (version 1.33m; National Institutes of Health, Bethesda, MD, USA) environment. The lengths of the medial gastrocnemius bellies were measured.

Each child's height was measured with a wall-mounted tape measure and corrected for any equinus and/or dynamic knee flexion deformity. For those who were incapable of standing upright independently, height was measured from the tip of the head to the heel while lying supine on the bed. Leg length was measured from the tibiofemoral cleft to the lateral malleolus.

We compared the difference in muscle lengths between similarly aged normal children and CP children.

The data from CP patients was used to measure the percentage of GM muscle length with leg length. The average of all the percentages can be used by operating surgeons to make more precise incisions.

### Ethical Approval

The study was approved by the Manchester Local Research Ethics Committee (MLREC) and the Manchester Metropolitan University Research Ethics Committee, in accordance with the Declaration of Helsinki.

## RESULTS

The participating children in both the groups were of similar ages (p>0.05). One of the participants with diplegia had knee flexion contracture of about 5°, and another participant had hip flexion of about 10° when lying prone. The alignment of the knee joint at rest was otherwise similar among the participants, and resting knee joint angle was excluded as a predictor variable in the subsequent analyses.

The median age in typically developing children was 8 years in females and 9 years in males (Table **1**). Similarly the median ages were 8 years and 9 years respectively in males and females suffering from hemi/ diplegia (Table **2**).

Table **3** shows that at same age the GM muscle lengths in CP patients are shorter than normal children (ranging between 3 to 9 cm).

$$\mathrm{Ratio}=\frac{\mathrm{GM}\text{\&nbsp;}\mathrm{Muscle}\text{\&nbsp;}\mathrm{Length}\text{\&nbsp;}\&times;100}{\mathrm{Leg}\text{\&nbsp;}\mathrm{Length}}$$

Table **5**. shows how accurate our formula is when compared to the actual measurement. For example the first female paretic child’s calculated muscle length using our formula is 12.3 cm, which is very close to the child’s actual muscle length of 12 cm. Whereas when compared to Tashjian *et al.* who postulated the ratio to be 50%, it can be seen that it’s gives a less accurate figure of 13.75 cm in the same child.

## DISCUSSION

CP may selectively affect the muscles in the lower limbs depending on the extent of the brain injury. It is commonly observed that only the gastrocnemius part of the triceps surae group of muscles in the lower leg is affected and may have increased tone or contractures. The treatment involves either injection of Botulinum toxin into the gastrocnemius muscle bellies to manage the spasticity or selective lengthening of the gastrocnemius aponeurosis [1]. Inaccurate injection of the Botulinum toxin or inappropriate lengthening of the tendon may lead to undesirable results.

The accurate localisation of muscle-tendon junction at the time of Botulinum injection or gastrocnemius slide (intramuscular lengthening) can be difficult. The palpation of the muscle bellies or previous experience of the operating surgeon is employed to place the surgical incision. An inappropriately long or short incision may result because of inaccurate localisation, along with a risk of iatrogenic damage to the vital structures *e.g.* sural nerve.

Elson *et al.* suggested that if the gastrocnemius tendon is to be exposed medially it would be reasonable to place a vertical skin incision between 38% and 46% of the distance between the upper border of the calcaneus and the fibular head [4]. It seems to have been based on the observation that the gastrocnemius muscle belly constitutes the proximal 38-46% of the triceps muscle tendon unit.

Tashjian *et al.* found that the gastrocnemius-soleus junction was located at approximately 50% of fibular length and Pinney *et al.* found that the site of surgical division of the gastrocnemius tendon was at an average of 43% of the length of the lower leg [1, 2].

The previous studies in English literature have used normal cadavers or *in-vivo* measurements (normal adults with gastrocnemius equinus contracture undergoing Strayer recessions) to localise the muscle-tendon junction [1-4]. It may be that these measurements of normal individuals are different in children with CP.

Table **3** clearly showed the measurements are significantly different even when compared with age matched CP and typically developing children.

The present study looked at the *in-vivo* gastrocnemius muscle-tendon relationship in patients with CP and compared it with a normal (unaffected) age and sex-matched group of children. It has provided an average figure based on the relationship of the gastrocnemius tendon to readily palpable anatomical landmarks in CP patients. The formula may be used clinically to identify the tendon for open or endoscopic lengthening.

A recent study by Kucharski et al demonstrated, that pre-operative ultrasound guided incision planning for gastrocnemius lengthening can accurately localise the muscle-tendon junction. However, this will increase the operating time and adds to increase resources needed in the theatre.

In the present study, the gastrocnemius muscle tendon junction ranged between 35 to 50%, a much wider range than described in earlier studies giving surgeons a wider range to work on. The average ratio was 44.9%, a much more precise figure. Table **5** clearly shows the accuracy of using this figure when compared with the real time observations. However, when comparing this figure to Tashjian *et al.* who postulated the ratio to be 50%, the figures are less accurate [1]. Using the 50% figure, would result in inaccurate localisation of gastrocnemius- soleus junction and more prone to iatrogenic injury.

## CONCLUSION

Knowledge of the relevant anatomy associated with the gastrocnemius muscle – tendon unit should allow surgeons to accurately target the planned treatment. This study has suggested that an incision centred at 45% of the lower leg length improves surgical access as well as decreasing the length of the surgical incision during gastrocnemius lengthening.

The new figure of 45% may be used in every day practice by orthopaedic surgeons to identify the muscle- tendon junction prior to open or endoscopic lengthening or botulinum toxin injection procedure.

## Figures and Tables

**Fig. (1):**
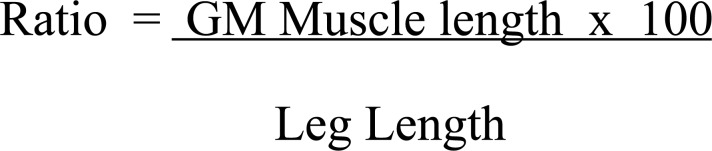
Formula for gastrocnemius medial muscle length: leg length ratio in CP.

**Table 1:** Demographics and measurements in typically developing children.

**Sex**	**Height(cm)**	**Age(yrs)**	**Leg Length** **(cm)**	**GM Muscle** **Length (cm)**	**GL Muscle** **Length (cm)**
**F**	**136**	**9**	**30.3**	**15.9**	**15.4**
**F**	**139.5**	**8**	**32**	**18.2**	**13.3**
**F**	**122**	**8**	**28.2**	**15.1**	**14.4**
**F**	**115**	**6**	**25.3**	**14.1**	**13**
**F**	**104.5**	**4**	**21.5**	**12**	**11.4**
**F**	**165**	**14**	**39**	**21.1**	**17.4**
**F**	**165**	**13**	**38.5**	**22.5**	**20**
	**Mean**	**8.9**	**30.7**	**17**	**15**
	**Median**	**8**	**30.3**	**15.9**	**14.4**
**M**	**117.5**	**7**	**25.6**	**17.7**	**14.8**
**M**	**139**	**9**	**33.2**	**20.4**	**19.3**
**M**	**152**	**13**	**37.2**	**18.2**	**16.8**
	**Mean**	**9.7**	**32**	**18.8**	**17**
	**Median**	**9**	**33.2**	**18.2**	**16.8**

Key: GM = Gastrocnemius medial, GL = Gastrocnemius lateral.

**Table 2:** Demographics and measurements of di/hemiplegic children.

				**GM** **Muscle**	**GL** **Muscle**
**Sex**	**Height**	**Age**	**Leg length**	**length**	**length**
**F**	**120**	**9**	**27.5**	**12**	**10.6**
**F**	**117**	**8**	**26**	**12.1**	**11.4**
**F**	**126.5**	**8**	**27**	**11.8**	**9.6**
**F**	**148**	**13**	**34**	**15**	**13.5**
**F**	**158**	**13**	**37**	**18.5**	**18.5**
**F**	**113.5**	**5.6**	**25**	**10.7**	**10.8**
**F**	**92**	**3**	**19.6**	**8.6**	**8.1**
	**Mean**	**8.7**	**28**	**12.7**	**11.9**
	**Median**	**8**	**27**	**12**	**10.8**
**M**	**80**	**2.8**	**17**	**8.3**	**7.8**
**M**	**127**	**9**	**26**	**13**	**12.8**
**M**	**157**	**14**	**38.8**	**13.7**	**11.6**
	**Mean**	**8.7**	**27.3**	**11.7**	**10.7**
	**Median**	**9**	**26**	**13**	**11.6**

Key: GM = Gastrocnemius medial, GL = Gastrocnemius lateral.

Typically developing children were significantly (p<0.05) taller (134.4cm [13.5] *vs* 121.4cm [19.2]) and heavier (32.7kg [10.7] *vs* 25.5kg [110]) than those with hemi/diplegia (Table **3**). They also had longer legs (31.0cm [3.9] *vs* 26.9cm [5.7])) and smaller resting ankle joint angles (25 ° [6] *vs* 38° [7]).

**Table 3:** Difference in GM muscle length between paretic and typically developing children.

**Non Paretic**		**Paretic**		
**Age**	**GM-Muscle** **length(cm)**	**Age**	**GM-Muscle** **length (cm)**	**Difference in length (cm)**
**4**	**12**	**3.1**	**8.6**	**3.4**
**6**	**14.1**	**5.6**	**10.7**	**3.4**
**7**	**17.7**	**2.8**	**8.3**	**9.4**
**8**	**18.2**	**8.3**	**12.1**	**6.1**
**8**	**15.1**	**8.3**	**11.8**	**3.3**
**9**	**15.9**	**9.3**	**12**	**3.9**
**9**	**20.4**	**9.3**	**13**	**7.4**
**13**	**22.5**	**13.1**	**18.5**	**4**
**13**	**18.2**	**13.4**	**15**	**3.2**
**14**	**21.1**	**14.1**	**13.7**	**7.4**

Our simple formula Fig. (**1**) can be used in di/hemiplegic patients to calculate the ratio/percentage of GM muscle length in comparison to the leg length (Table **4**). The average of these ratios is 44.9%.

**Table 4:** The formula in Fig. (**1**) is used in di/hemiplegic patients to calculate the percentage of GM muscle length in comparison to the leg length.

**Age**	**GM Muscle length (cm)**	**Leg length**	**% Leg length**
**3.1**	**8.6**	**19.6**	43.9
**5.6**	**10.7**	**25**	42.8
**2.8**	**8.3**	**17**	48.8
**8.3**	**12.1**	**26**	46.5
**8.3**	**11.8**	**27**	43.7
**9.3**	**12**	**27.5**	43.6
**9.3**	**13**	**26**	50
**13.1**	**18.5**	**37**	50
**13.4**	**15**	**34**	44.1
**14.1**	**13.7**	**38.8**	35.3

We used this round figure of 45% when calculating GM muscle length. The surgeon will measure leg length from the tibiofemoral joint line to the lateral malleolus. Multiplying the measured length with 0.45% will calculate GM muscle length.

**Table 5:** Actual vs calculated muscle length.

		**Actual**	**Calculated**	**Tashjian *et al.*** **(50% GM-Muscle**
**Sex**	**Lower leg length**	**GM-Muscle length**	**GM-Muscle length**	**length)**
**F**	**27.5**	**12**	**12.375**	**13.75**
**F**	**26**	**12.1**	**11.7**	**13**
**F**	**27**	**11.8**	**12.15**	**13.5**
**F**	**34**	**15**	**15.3**	**17.5**
**F**	**37**	**18.5**	**16.65**	**18.5**
**F**	**25**	**10.7**	**11.25**	**12.5**
**F**	**19.6**	**8.6**	**8.82**	**9.8**
**M**	**17**	**8.3**	**7.65**	**8.5**
**M**	**26**	**13**	**11.7**	**13**
**M**	**38.8**	**13.7**	**17.46**	**19.4**

## LIST OF ABBREVIATIONS:

CP: = Cerebral Palsy.
GM: = Gastrocnemius medialis.
GL: = Gastrocnemius lateralis.

## References

[1] Tashjian R.Z., Appel A.J., Banerjee R., DiGiovanni C.W. (2003). Anatomic study of the gastrocnemius-soleus junction and its relationship to the sural nerve.. Foot Ankle Int..

[2] Pinney S.J., Sangeorzan B.J., Hansen S.T. (2004). Surgical anatomy of the gastrocnemius recession (Strayer procedure).. Foot Ankle Int..

[3] Carl T., Barrett S.L. (2005). Cadaveric assessment of the gastrocnemius aponeurosis to assist in the pre-operative planning for two portal endoscopic gastrocnemius recession (EDR).. Foot.

[4] Elson D.W., Whiten S., Hillman S.J., Johnson R.J., Lo S.S., Robb J.E. (2007). The conjoint junction of the triceps surae: Implications for gastrocnemius tendon lengthening.. Clin. Anat..

[5] Kucharski R.A., Campbell D., Bell M. http://online.boneandjoint.org.uk/doi/pdf/10.1007/s11832-009-0162-0.

[6] Mohagheghi A.A., Khan T., Meadows T.H., Giannikas K., Baltzopoulos V., Maganaris C.N. (2008). *in vivo* gastrocnemius muscle fascicle length in children with and without diplegic cerebral palsy.. Dev. Med. Child Neurol..

